# Ubiquitin E3 Ligase Ring1b/Rnf2 of Polycomb Repressive Complex 1 Contributes to Stable Maintenance of Mouse Embryonic Stem Cells

**DOI:** 10.1371/journal.pone.0002235

**Published:** 2008-05-21

**Authors:** Petra van der Stoop, Erwin A. Boutsma, Danielle Hulsman, Sonja Noback, Mike Heimerikx, Ron M. Kerkhoven, J. Willem Voncken, Lodewyk F. A. Wessels, Maarten van Lohuizen

**Affiliations:** 1 Division of Molecular Genetics, The Netherlands Cancer Institute, Amsterdam, The Netherlands; 2 Center for Biomedical Genetics, The Netherlands Cancer Institute, Amsterdam, The Netherlands; 3 Bioinformatics and Statistics, Division of Molecular Biology, The Netherlands Cancer Institute, Amsterdam, The Netherlands; 4 Department of Molecular Genetics, Maastricht University Medical Centre, Maastricht, The Netherlands; 5 Department of Neurogenetics, Academic Medical Center, University of Amsterdam, Amsterdam, The Netherlands; Texas Tech University Health Sciences Center, United States of America

## Abstract

**Background:**

Polycomb repressive complex 1 (PRC1) core member Ring1b/Rnf2, with ubiquitin E3 ligase activity towards histone H2A at lysine 119, is essential for early embryogenesis. To obtain more insight into the role of Ring1b in early development, we studied its function in mouse embryonic stem (ES) cells.

**Methodology/Principal Findings:**

We investigated the effects of Ring1b ablation on transcriptional regulation using *Ring1b* conditional knockout ES cells and large-scale gene expression analysis. The absence of Ring1b results in aberrant expression of key developmental genes and deregulation of specific differentiation-related pathways, including TGFbeta signaling, cell cycle regulation and cellular communication. Moreover, ES cell markers, including *Zfp42*/*Rex-1* and *Sox2*, are downregulated. Importantly, retained expression of ES cell regulators Oct4, Nanog and alkaline phosphatase indicates that Ring1b-deficient ES cells retain important ES cell specific characteristics. Comparative analysis of our expression profiling data with previously published global binding studies shows that the genes that are bound by Ring1b in ES cells have bivalent histone marks, i.e. both active H3K4me3 and repressive H3K27me3, or the active H3K4me3 histone mark alone and are associated with CpG-‘rich’ promoters. However, deletion of Ring1b results in deregulation, mainly derepression, of only a subset of these genes, suggesting that additional silencing mechanisms are involved in repression of the other Ring1b bound genes in ES cells.

**Conclusions:**

Ring1b is essential to stably maintain an undifferentiated state of mouse ES cells by repressing genes with important roles during differentiation and development. These genes are characterized by high CpG content promoters and bivalent histone marks or the active H3K4me3 histone mark alone.

## Introduction

PcG proteins have initially been identified in *Drosophila* as transcriptional repressors required for correct expression of homeotic (*Hox*) genes. By means of chromatin remodeling, PcG proteins maintain stable gene repression to ensure proper embryonic development. In mammals, two biochemically and functionally distinct PcG core complexes have been identified [1]. Mammalian polycomb repressive complex 1 (PRC1) consists of close homologs of the *Drosophila* PRC1 core members Ph, Pc, Psc and dRing [1], [2]. Ring1b/Rnf2, the mouse homolog of dRing, acts as a ubiquitin E3 ligase towards histone H2A at lysine 119 resulting in mono-ubiquitinated H2A (uH2A) [3], [4]. Ezh2/Kmt6, a methyltransferase that trimethylates histone H3 at lysine 27 (H3K27me3), acts in complex with Suz12 and Eed, constituting Polycomb repressive complex 2 (PRC2) [5]–[7]. PRC1 and PCR2 do not physically interact, but the Ezh2 catalyzed histone mark H3K27me3 is recognized by PRC1 member Pc, providing a mechanism of communication between the two complexes [8]. Furthermore, PRC1 binding to chromatin requires PRC2, although PRC2-independent recruitment of PRC1 is also reported [9], [10]. The counteracting trithorax group (TrxG) proteins mediate an active transcriptional state by methylation of histone H3 at lysine 4 (H3K4me3) [2].

Proper expression of PRC2 proteins is essential during early embryonic development,, since mice lacking one of these proteins die due to gastrulation defects [11]–[13]. In contrast, PRC1 members appear to be more important during later stages of development, with the clear exception of Ring1b, which evokes an early embryonic lethal phenotype similar to PRC2 null mice [14]–[18]. PcG proteins were shown to be important for self-renewal and maintaining pluripotency of ES cells [19]–[21]. ES cells are derived from the inner cell mass of pre-implantation blastocysts [22]. ES can self-renew and maintain a pluripotent, undifferentiated state under the right conditions *in vitro*, while retaining the capacity to differentiate into every cell type required during development *in vitro* and *in vivo* when reintroduced back into a host blastocyst [23]. Genome-wide and candidate-based studies revealed that PcG proteins maintain this undifferentiated state of ES cells through direct repression of developmental genes [21], [24]–[27]. Developmental genes are largely associated with bivalent (i.e. both repressive H3K27me3 and active H3K4me3) histone marks and are silent or expressed at very low levels in ES cells [28]–[30]. Bivalent domains tend to resolve during development [28]–[30]. Therefore, it is suggested that this bivalent chromatin state poises genes for transcriptional activation (loss of H3K27me3) or prolonged repression (loss of H3K4me3 or both marks) during later developmental stages. Only recently, it was shown that Ring1b-mediated uH2A deposition at repressed bivalent genes restrains a poised RNA polymerase II (RNAPII) configuration [31]. Conditional deletion of Ring1b and subsequent loss of uH2A from the promoter and coding region results in release of poised RNAPII and gene derepression. More evidence for a direct role of uH2A in negative transcriptional regulation was found by another group, which investigated the role of histone H2A E3 ligase 2A-HUB in repressing chemokine genes in human monocytes [32]. They show that the presence of uH2A in the promoter-proximal region physically blocks recruitment of FACT, thereby causing RNAPII-dependent transcription elongation to pause.

To explore the role of Ring1b in regulation of gene expression during early development, we analyzed genome-wide changes in gene transcription following deletion of Ring1b in ES cells. Hereto, we employed an inducible knockout system and performed a genome-wide screen using microarrays to identify genes governed by Ring1b. We identify several processes and pathways differentially regulated in Ring1b-deficient ES cells, which are implicated in differentiation and embryogenesis. Importantly, we find that Ring1b-deficient ES cells retain expression of key stem cell regulators Oct4 and Nanog. Finally, by comparing our expression data with previously published global binding studies, we find that Ring1b preferentially represses genes with high CpG-content promoters and bivalent or H3K4me3 histone marks. This suggests that Ring1b has an important role in maintaining an undifferentiated state of ES cells. Furthermore, this study provides more insight in the characteristics of the genes that are under direct transcriptional control of Ring1b in ES cells.

## Results

### Generating *Ring1b* conditional knockout mouse embryonic stem cells

**Figure 1:**
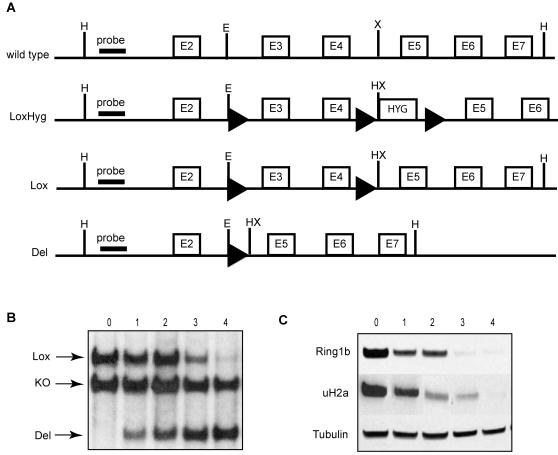
Generating *Ring1b* conditional knockout ES cells. (A) Schematic overview of the *Ring1b* locus targeting strategy in mouse *Ring1b^+/−^* ES cells. The wild type *Ring1b* allele is shown with main restrictions sites, introns and exons, in the targeted genomic region. The *Ring1b^LoxHy^* ^g^ allele was generated by targeting a floxed exon3+4/Hygromycin replacement vector to the wild type *Ring1b* locus of the *Ring1b^+/−^* ES cells. The hygromycin cassette allowed for selection after the initial targeting and was removed by transiently expressing adenoviral Cre to generate the conditional *Ring1b^Lox^* allele. Acute loss of Ring1b from *Ring1b^Lox^* ES cells was induced by adding 4-OHT to the medium to activate CreER^T2^ (Cre-recombinase fused to a mutated ligand binding domain of the human estrogen receptor (ER), targeted to the ROSA locus, not shown) resulting in deletion of exons 3 and 4, represented as the *Ring1b^Del^* allele. Exons are indicated by white boxes with the corresponding exon number, loxP sequences by black triangles, and HYG indicates the hygromycin cassette. Main restriction sites are indicated (H = HindIII, E = EcoRI, X = XhoI) as well as the location of the 5′end probe used for Southern blot analysis. (B) Southern blot analysis of HindIII digested genomic DNA of *Ring1b^−/Lox^;CreER^T2^* ES cells showing recombination of the *Ring1b^Lox^* allele following treatment with 4-OHT for the indicated time in days. The 5′end probe visualizes the conventional knockout allele (5 kb), the conditional *Lox* allele (7 kb) and the recombined *Del* allele (4 kb). (C) Western blot analysis showing loss of Ring1b protein and uH2A in *Ring1b^−/Lox^;CreER^T2^* ES cells following treatment with 4-OHT for the indicated time in days. Tubulin serves as a loading control.

To gain insight in the role of Ring1b in ES cells, we generated *Ring1b* conditional knockout mouse ES cells. Previously, we have shown that deletion of the RING finger domain of Ring1b, encoded by exon 3 and 4, generates a functional null allele [17]. *Ring1b* conditional knockout (Ring1b^−/Lox^) ES cells were then generated by a second targeting round in *Ring1b^+/−^* ES cells [17] using a targeting vector, which introduced loxP sequences flanking these exons (Figure 1A; details in Materials and Methods). To generate inducible *Ring1b^−/Lox^* ES cells, we targeted a 4-hydroxytamoxifen (4-OHT) inducible R26CreER^T2^ construct (Cre-recombinase fused to a mutated ligand binding domain of the human estrogen receptor) to the ROSA26 locus of *Ring1b^−/Lox^* ES cells, hereafter named *Ring1b^−/Lox^;CreER^T2^* ES cells. The presence of loxP sequences in the genomic locus of Ring1b did not alter the expression of Ring1b in ES cells. Accordingly, *Ring1b^−/Lox^* and *Ring1b^−/Lox^;CreER^T2^* mice developed indistinguishable from wild type mice (E.B, M.v.L, unpublished results).

Addition of 4-OHT to the culture medium resulted in recombination of the *Ring1b* conditional allele in *Ring1b^−/Lox^;CreER^T2^* ES cells and subsequent depletion of Ring1b protein as was shown by Southern and Western blot analysis (Figure 1B, 1C). We observed cellular detachment and apoptosis concurrent with an increase in cells with a differentiated phenotype after deleting Ring1b in *Ring1b^−/Lox^;CreER^T2^* ES cells, but not in similarly treated *Ring1b^+/+^;CreER^T2^* control ES cells. Based on Annexin V staining followed by flow cytometric analysis, about 83% of all *Ring1b^−/Lox^;CreER^T2^* ES cells were undergoing apoptosis four days following 4-OHT treatment (data not shown). Of the adherent population only 15% was Annexin V positive, compared to 5% of the untreated population (data not shown). We therefore focused our studies on the immediate early effects of the first days following Cre-mediated deletion of Ring1b in the viable, adherent ES cells.

### Ring1b is required for mono-ubiquitination of H2A in ES cells

**Figure 2:**
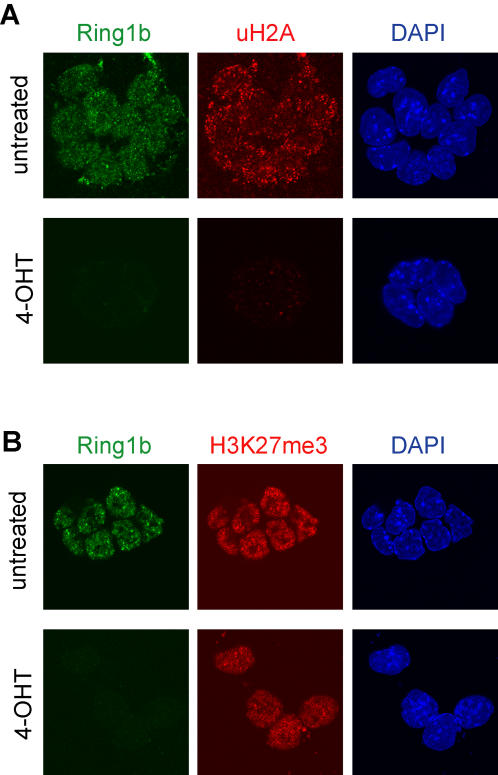
Deletion of Ring1b results in loss of uH2A but not H3K27me3. Examples of immunofluorescence stainings in *Ring1b^−/Lox^;CreER^T2^* ES cells showing loss of uH2A (A), but not H3K27me3 (B) in cells that have lost Ring1b four days following 4-OHT treatment.

Since Ring1b is a ubiquitin E3 ligase for histone H2A at lysine 119, we studied the effect of Ring1b deletion on the levels of uH2A. Western blot analysis showed that uH2A levels decreased to almost undetectable levels after deletion of Ring1b from *Ring1b^−/Lox^;CreER^T2^* ES cells (Figure 1C). In addition, immunofluorescence stainings confirmed that uH2A was lost in ES cells that had lost Ring1b expression (Figure 2A). The same held true for mouse embryonic fibroblasts (MEFs) derived from *Ring1b^−/Lox^;CreER^T2^* mouse embryos, which showed no uH2A immunofluorescence staining in Ring1b-negative cells (Figure S1). Immunofluorescence stainings for H3K27me3, the polycomb repressive mark set by PRC2 member Ezh2, showed that this mark was present in all cells (Figure 2B). These results confirm that Ring1b is the main E3 ligase for mono-ubiquitination of H2A in both mouse ES cells and MEFs and suggest that deletion of Ring1b does not affect H3K27 trimethylation.

### Ring1b deficiency affects expression of other PRC1 core members

**Figure 3:**
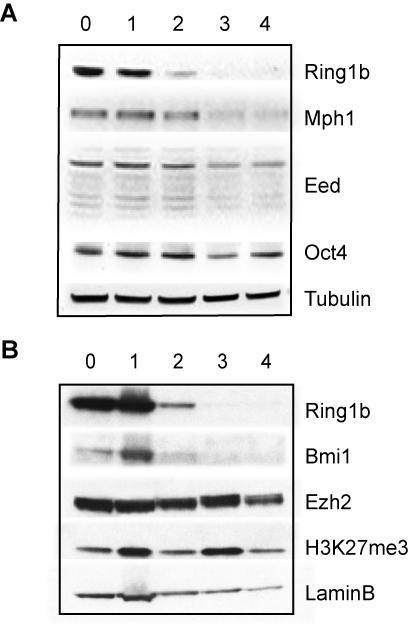
Downregulation of protein levels of PRC1, but not PRC2, following Ring1b deletion in ES cells. Western blot analysis of total cell protein extracts (A) or nuclear extracts (B) of *Ring1b^−/Lox^;CreER^T2^* ES cells shows that deletion of Ring1b results in downregulation of Phc1/Mph1 and Bmi1 protein levels, but not of Ezh2, Eed or H3K27me3 levels following treatment with 4-OHT for the indicated number of days.

Studies in *Drosophila,* mouse and human cells have shown that PcG genes themselves are PcG protein binding targets, suggesting auto-regulation within the PcG family [21], [25], [33]. We therefore examined the effect of Ring1b deletion on other PRC1 and PRC2 members in ES cells. Microarray analysis (described in more detail in the following section) showed that the transcript levels of *Ring1b* and *Phc1/Mph1* were significantly downregulated over time, and correspondingly decreased in protein levels (Figure 3A, Table S1). Interestingly, PRC1 member *Bmi1* was derepressed transcriptionally in the absence of Ring1b, but its protein levels were down-regulated, suggesting that Bmi1 is post-transcriptionally regulated by Ring1b (Figure 3B). This is consistent with observations by others [34], and may be explained by the fact that interaction between Bmi1 and Ring1b protects both proteins from ubiquitin-mediated degradation [35]. The effect of loss of Ring1b on other PRC1 core members may explain the severity of the Ring1b mouse knockout phenotype compared to knockout models of other PRC1 members [14]–[18]. Other polycomb group genes of which the transcription had increased significantly were *Epc1*, *Phf1*, and *Phc2* (Table S1). No effects were observed on the protein or transcript levels of PRC2 members *Ezh2* and *Eed*, or the levels of H3K27me3, which is in agreement with the immunofluorescence analysis (Figure 3A, 3B, Table S1). The effect on PcG protein and transcript levels after loss of Ring1b is in agreement with an earlier study performed in established Ring1b-deficient ES cells, with the exception of the transcriptional downregulation of Phc1 [34]. This might therefore reflect an indirect effect of acute loss of Ring1b in ES cells. These results suggest that deletion of Ring1b affects the transcript and protein levels of members of PRC1, but not of PRC2, and underscores the importance of Ring1b for PRC1 stability.

### Regulation of developmental processes through derepression of Ring1b bound genes

**Figure 4:**
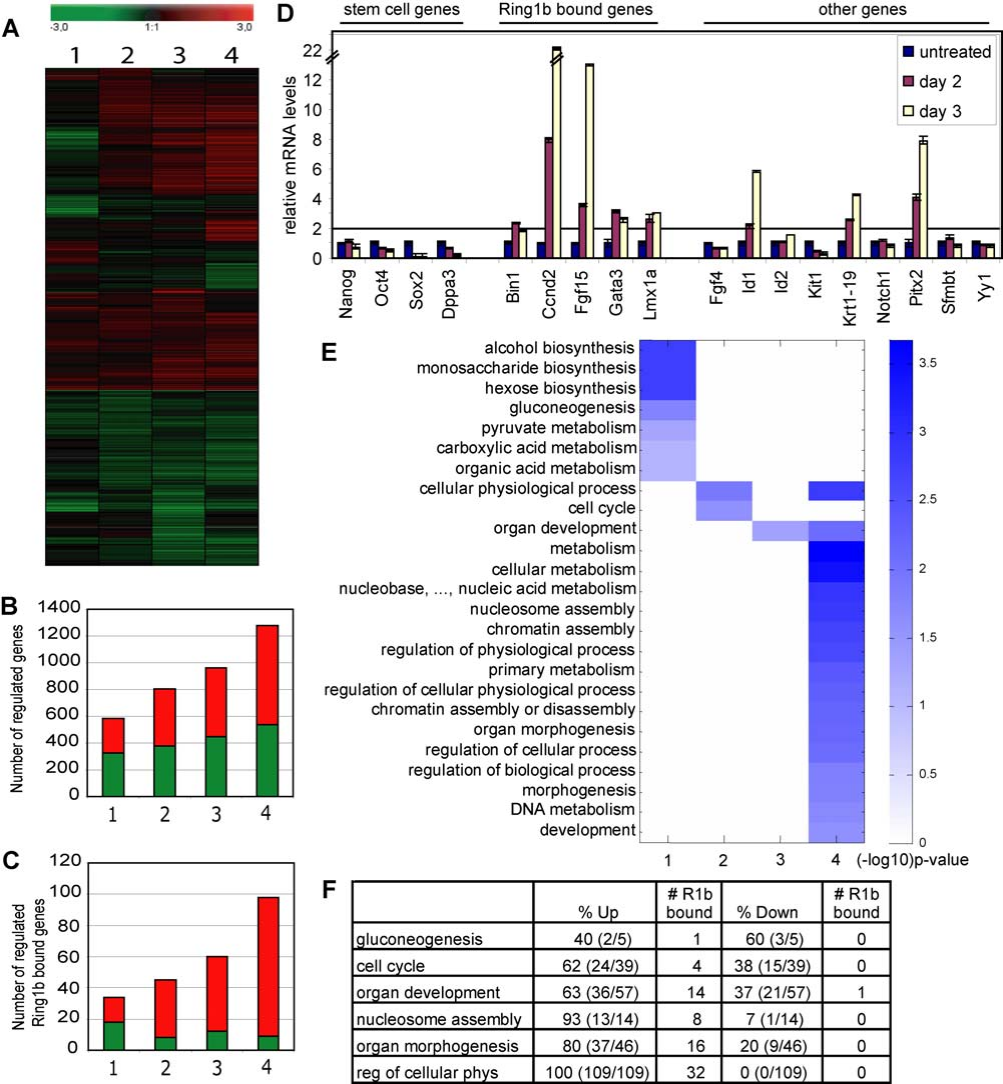
Gene expression profiling in Ring1b-deficient ES cells. (A) Expression profiling data clustered by the software program Genesis shows regulation of gene expression (p<0.05) after deletion of Ring1b in mouse ES cells over time. (B) Graph representing the number of all upregulated (red) and downregulated (green) genes (p<0.05). (C) Graph representing the number of up- (red) or downregulated (green) Ring1b bound genes specifically [21]. (D) QPCR validation of microarray data for a selection of genes. Time indicates number of days of 4-OHT treatment. (E) Heatmap of the statistically overrepresented GO categories, as identified by BiNGO analysis. The color scale bar represents the relation between the color intensity and the level of significance (–log10(p-value)). (F) Table showing the significantly enriched GO categories and the percentage (numbers between brackets) of up- or downregulated genes and the number of Ring1b bound genes per GO category (the ones most-downstream in the GO-tree, see also Figure S2).

To obtain insight in the genome-wide effects on gene transcription after deletion of Ring1b in ES cells, we performed microarray analysis. We analyzed the temporal changes in gene transcription in *Ring1b^−/Lox^;CreER^T2^* ES cells one to four days after deletion of Ring1b. As a reference, we used *Ring1b^+/+^;CreER^T2^* ES cells similarly treated with 4-OHT to correct for any effects induced by 4-OHT or CreER^T2^. We identified 2365 differentially regulated genes, i.e. genes that were significantly (p<0.05 according to the Rosetta-error model, for details see Materials and Methods) up- or downregulated (7%; 2365/31769; Figure 4A, 4B, Table S1). The reliability of the microarray data was confirmed by quantitative real-time PCR analysis (QPCR) on a selection of 18 genes (Figure 4D).

To gain insight into the processes that are deregulated in absence of Ring1b in mouse ES cells, we analyzed the deregulated genes based on their ontology classification. To this end, we employed the gene ontology (GO) tool BiNGO to determine which GO categories are statistically overrepresented. By mapping the predominant functional GO categories in a hierarchical graph BiNGO facilitates insight in the hierarchical structure and relations between the ontologies [36]. We found that the outliers were involved in various processes, of which the most downstream in the GO-tree were: ‘organ development’, ‘organ morphogenesis’, ‘nucleosome assembly’, ‘regulation of cellular physiological processes’, ‘glyconeogenesis’, and ‘cell cycle’ (Figure 4E; for a graphical overview of the overrepresented GO categories, see Figure S2; for the BiNGO analysis, see Table S2). These data confirm that Ring1b is involved in regulating cell cycle, developmental and chromatin-related processes and indicate that deletion of Ring1b affects glyconeogenesis and the regulation of cellular physiological processes. Given that loss of Ring1b affects the undifferentiated state of ES cells, the regulated processes are likely to include genes that are deregulated due to indirect effects.

By using a genome-wide ChIP-on-chip analysis Boyer and colleagues identified genes that are bound by Ring1b in mouse ES cells [21]. By combining our microarray expression data with the data from this Ring1b binding study, we were able to determine which genes were direct transcriptional targets of Ring1b. Of the 1219 Ring1b bound genes, identified by Boyer and colleagues, 1078 were represented on our microarray. Remarkably, we found that only a subset of 141 Ring1b bound genes had significantly changed expression (13% (141/1078). These genes were predominantly upregulated, which is in agreement with Ring1b being a transcriptional repressor (Figure 4C). The partial derepression implies that other regulatory mechanisms are active at the other Ring1b target genes in addition to Ring1b, and reflects that a subset of genes is deregulated due to indirect effects of loss of Ring1b in ES cells.

Next, we assessed which of the deregulated genes within an overrepresented GO category (most down-stream in the GO-tree, see also Figure S2) were bound by Ring1b. We found that on average 30% of the deregulated genes within an enriched ontology was bound by Ring1b, in particular in the ontologies organ development and morphogenesis, nucleosome assembly, and regulation of cellular physiological process (Figure 4F). This was substantially higher than what would be expected considering that only 6% (141/2365) of all deregulated genes was bound by Ring1b. This indicates that Ring1b is involved in direct transcriptional regulation of a substantial large fraction of the deregulated genes in these ontologies. Together, these data suggest that Ring1b is specifically important for dynamic repression of developmental, chromatin-related, cell cycle and metabolic genes in ES cells.

### Regulation of specific differentiation-related pathways in Ring1b-deficient ES cells

The analysis based on the ontology classification shows that absence of Ring1b results in aberrant expression of genes involved in developmental and various cellular processes. We set out to identify specific pathways that are affected in Ring1b-deficient ES cells. To remain unbiased in the analysis of our set of outliers, we used the bioinformatics tool DAVID, which uses information of well-defined pathways available in the KEGG pathway database (http://david.abcc.ncifcrf.gov). Interestingly, we found that the identified pathways have roles in development and differentiation of ES cells. A selection is listed in Table 1. A complete overview of the pathway analysis can be found in Table S3.

**Table 1:** A selection of the deregulated genes and corresponding pathways affected in Ring1b-deficient ES cells.

Symbol	EntrezID	Regulation	bound	Symbol	EntrezID	Regulation	bound
**Focal Adhesion**				**TGFbeta signaling**			
*Actg2*	11464	up	0	*Amhr2*	110542	down	ND
*Actn3*	11474	down	0	*Bmp7*	12162	up	1
*Capn2*	12334	up	0	*Bmpr1a*	12166	up	0
*Capn5*	12337	down	0	*Cul1*	26965	up	0
*Ccnd1*	12443	up	0	*Id1*	15901	up	0
*Ccnd2*	12444	up	1	*Id2*	15902	up	0
*Col4a2*	12827	up	1	*Id3*	15903	up	1
*Grb2*	14784	down	0	*Lefty1*	13590	up	0
*Ilk*	16202	up	0	*Mapk3*	26417	down	ND
*Nras*	18176	down	0	*Nodal*	18119	up	0
*Pdgfc*	54635	up	ND	*Pitx2*	18741	up	0
*Pten*	19211	up	0	*Thbs1*	21825	up	0
*Rras2*	20130	down	0				
*Shc2*	216148	down	ND	**Cell cycle**			
*Thbs1*	21825	up	0	*Anapc10*	68999	up	0
*Vegfa*	22339	down	0	*Ccnd2*	12444	up	1
*Zyx*	22793	up	0	*Cdc20*	107995	up	0
				*Cdc27*	217232	up	0
**Gap junction**				*Cdc7*	12545	down	0
*Itpr1*	16438	down	0	*Cdkn1c*	12577	up	1
*Adcy2*	210044	up	1	*Chek1*	12649	down	0
*Csnk1d*	104318	up	0	*Cul1*	26965	up	0
*Edg2*	14745	down	0	*Gadd45a*	13197	down	0
*Gja1*	14609	up	1	*Gadd45g*	23882	up	1
*Grb2*	14784	down	0	*Mcm6*	17219	down	0
*Nras*	18176	down	0	*Sfn*	55948	up	ND
*Pdgfc*	54635	up	ND	*Smc1l2*	140557	down	0
*Rras2*	20130	down	0				
**Adherens junctions**							
*Actn3*	11474	down	0				
*Baiap2*	108100	up	0				
*Iqgap1*	29875	up	0				
*Lmo7*	380928	down	ND				
*Mapk3*	26417	down	ND				
*Pvrl4*	71740	down	0				
*Vcl*	22330	down	0				
*Wasl*	73178	up	0				

DAVID Pathway analysis revealed the pathways deregulated in Ring1b-deficient ES cells. Shown are the deregulated genes per pathway, their EntrezIDs, the deregulated expression state, and binding of Ring1b to the promoter (1 = bound, 0 = not bound, ND = no data). For a complete overview of all identified pathways see Table S3.

Most revealing was the identification of the TGFbeta signaling pathway of which Bmp/Id signaling (derepressed *Bmp7*, *Bmpr1a*, *Id1*, *Id2*, and *Id3*) and Nodal/Pitx2 signaling (derepressed *Nodal*, *Pitx2*) were suggested to be transcriptionally upregulated upon loss of Ring1b. Genome-wide binding studies have identified that PcG proteins are associated with members of the TGFbeta signaling pathway and show PcG dissociation from the genes that were derepressed following differentiation [21], [25]. These data support a role for Ring1b/PcG in direct repression of TGFbeta signaling in ES cells.

In agreement with the GO classification analysis, we found altered expression of several components of the cell cycle pathway. Changes in cell cycle regulation have been observed following differentiation of ES cells, characterized by amongst others activation of the pRb-E2f controlled G1 checkpoint, which is inoperative in ES cells [37], [38]. In line with this, key cell cycle regulators involved in activation of the G1/S checkpoint (derepressed *Cdkn1c*; downregulated *E2f1*, *Cdc7* and *Mcm6*) were transcriptionally deregulated. However, deregulation of other key cell cycle genes suggest promotion of S phase (derepressed *Ccnd2/Cyclin D2* and *Cul1*), G2/M arrest (derepressed *Gadd45g* and *Sfn*/*14-3-3-sigma*) or bypass of G2/M arrest (downregulated *Gadd45a*) and mitotic exit (derepressed *Cdc20* and *Cdc27*). This could imply that Ring1b has a dual role in cell cycle regulation in ES cells, which is supported by the observation that Ring1b is involved in direct repression of cell cycle promoting gene *Ccnd2* and cell cycle inhibitory genes *Cdkn1c* and *Gadd45g*.

Various components of cellular communication pathways were deregulated in the absence of Ring1b. These comprise ECM genes (derepressed *Col4a2* and *Thbs1*) involved in focal adhesion, gap junction genes (derepressed *Gja1* and *Csnk1d* and downregulated *Itpr1*), and adherens junction pathways, which are linked to the actin cytoskeleton organization (downregulated *Vcl* and *Actn3*, derepressed *Iqgap1*). Several of these genes were reported to change expression during differentiation and development [39]–[41].

Summarizing, this suggests that Ring1b deficiency induces changes in cellular pathways, such as Bmp/TGFbeta signaling, cell cycle regulation, and cellular communication, which strongly suggests a link to differentiation programs in ES cells.

### Retained expression of stem cell regulators in Ring1b-deficient ES cells

**Figure 5:**
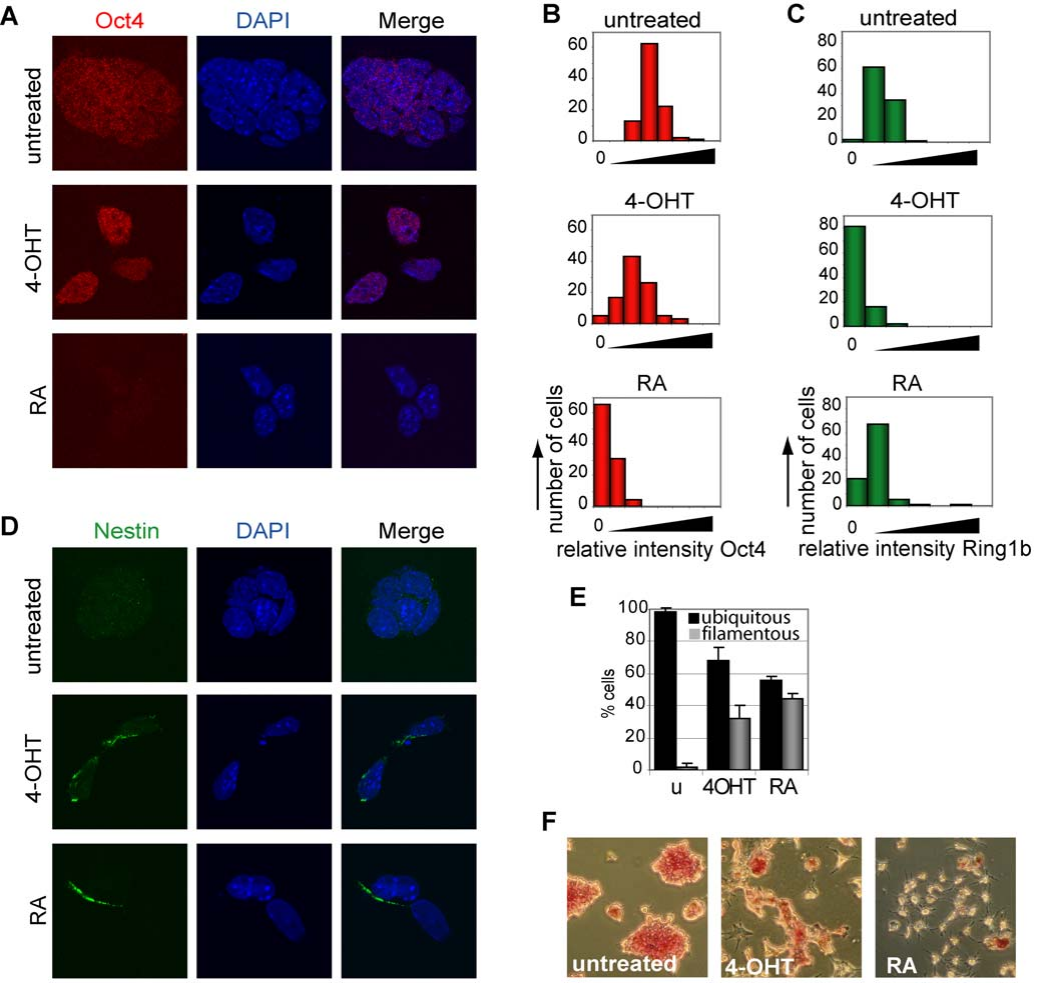
Ring1b-deficient ES cells retain expression of stem cell specific regulators. (A,D) Examples of immunofluorescence stainings in *Ring1b^−/Lox^;CreER^T2^* ES cells of (A) Oct4 (red) and DAPI (blue), and (D) Nestin (green) and DAPI (blue), either untreated (upper panels) or treated for four days with 4-OHT (middle panels) or three days with RA (lower panels). (B,C) Histograms represent the distribution of the mean immunofluorescence levels per cell retrieved after quantification of immunofluorescence stainings of Oct4 (B) or Ring1b (C) in *Ring1b^−/Lox^;CreER^T2^* ES cells either untreated, or treated for four days with 4-OHT or three days with RA. (E) Graph representing the number of *Ring1b^−/Lox^;CreER^T2^* ES cells either untreated, or treated for four days with 4-OHT or three days with RA that show ubiquitous (black bars) or filamentous (grey bars) Nestin staining. (F) Alkaline phosphatase activity stainings in *Ring1b^−/Lox^;CreER^T2^* ES cells either untreated, or treated for four days with 4-OHT or three days with RA.

ES cells, derived from the epiblast of early blastocysts, can self-renew and maintain a pluripotent, undifferentiated state *in vitro* by extrinsic factors, such as LIF and Bmp, and intrinsic factors, including stem cell regulators Oct4 and Nanog [42]–[45]. Upon induction of lineage-specific genes, these stem cell regulators are downregulated through a negative-feedback-loop by not yet understood mechanisms [42], [45], [46]. In line with this, the transcript levels of stem cell specific genes *Sox2*, *Dppa3*/*Stella* and *Zfp42*/*Rex-1* were strongly downregulated in Ring1b-deficient ES cells [43], [47] (Figure 4D, Table S1). However, QPCR analysis showed that *Nanog* transcript levels had not altered and *Oct4* transcript levels had reduced only to about 55% (Figure 4D). Importantly, *Oct4* was not identified as an outlier on the microarray and Western blot analysis showed no significant changes in Oct4 protein levels (Figure 3A, Table S1). To examine Oct4 expression in more detail, we also quantified the immunofluorescence levels of Oct4 and Ring1b stainings in individual cells. We compared this to guided neural differentiation induced by retinoic acid (RA) treatment, which was demonstrated to result in significant downregulation of Oct4 [48]. As expected, we found that three days after RA induced neural differentiation Oct4 was completely downregulated, in about 65% of the cells (Figure 5A, 5B). However, whereas four days following Ring1b deletion, 81% of all cells were negative for Ring1b, only 5% had lost Oct4 expression (Figure 5A, 5B, 5C). To assess the extent of neural differentiation, we next examined the expression of neuronal-lineage marker Nestin [49]. We found that three days after RA treatment approximately 44% of the cells showed filamentous Nestin staining, compared to 32% of the cells after deletion of Ring1b (Figure 5D, 5E). This is remarkable considering the number of cells expressing Oct4. This suggests that expression of lineage markers co-exists with stem cell regulators in absence of Ring1b in ES cells. Finally, we examined the activity of alkaline phosphatase, which is highly expressed in undifferentiated, pluripotent ES cells [50]. We found that after deleting Ring1b a substantial number of cells still showed high activity of alkaline phosphatase compared to RA treated ES cells, which is indicative for a pluripotent, undifferentiated state of ES cells (Figure 5F). Combined, our findings suggest that Ring1b-deficient ES cells show features of differentiated cells, while retaining important ES cell characteristics.

### Ring1b represses genes that are co-occupied by stem cell regulators Oct4 and Nanog in ES cells

The stem cell-specific transcription factors Oct4 and Nanog control ES cell pluripotency by regulating expression of stem cell specific genes and repression of differentiation-specific genes, of which a subset is co-occupied by PcG proteins [25], [43], [51]. Considering the retained expression of Oct4 and Nanog, we were interested to see the effect on gene transcription of these co-occupied genes in absence of Ring1b. We therefore analyzed the changes in expression of genes that were bound by Ring1b [21] and by Oct4 and/or Nanog in mouse ES cells as was identified by Loh and co-workers [51]. We found that the expression of 25 of the 212 Nanog/Oct4/Ring1b bound genes represented on our microarray was altered in Ring1b-deficient ES cells (Figure 4D, Table S1). We found that 18 of these 25 genes were derepressed, including *Fgf15*, *Bmp7*, *Gata3*, *Bmi1*, *Msx2*, *Podxl*, *Col4a2*, *Gadd45g*, *Gja1* and *Eif4g3*. This suggests that Ring1b is required to repress a specific subset of Oct4 and Nanog bound genes to maintain a pluripotent, undifferentiated ES cell state.

### Ring1b represses bivalent or H3K4me3 marked genes with CpG-rich promoters

Ring1b-mediated H2A mono-ubiquitination was shown to restrain a poised RNAPII configuration specifically at the promoters of bivalent genes, which were derepressed upon loss of Ring1b and uH2A [31]. Therefore, we investigated the chromatin state of the genes that were deregulated in absence of Ring1b in ES cells. Hereto, we used a data set from Mikkelsen and colleagues, who generated genome-wide chromatin-state maps for mouse ES cells, neural progenitor cells (NPC) and MEFs [30]. Of 948 of the 1078 Ring1b bound genes [21] represented on our microarray chromatin-state maps were retrieved for the histone modifications H3K4me3 and H3K27me3 [30]. Notably, virtually all (99%) of the Ring1b bound genes that were mapped as marked with both H3K27me3 and H3K4me3 or H3K27me3 alone, according to Mikkelsen-data set [30], were associated with H3K27me3 according to the H3K27me3 mapping data by Boyer et al [21], indicating a strong overlap between the two data sets for this histone mark. The chromatin-state maps also contain information about the CpG content of promoters genome-wide. Mikkelsen and colleagues show that genes with high-CpG content promoters (HCP) and H3K4me3 marks generally have ‘housekeeping’ functions, including replication and basic metabolism, while genes with HCP and bivalent marks mainly include key developmental transcription factors, morphogens, and cell surface markers. Genes with low-CpG content promoters (LCP) are associated with highly tissue-specific genes. A third class of genes with intermediate CpG promoter content (ICP) was added to ensure discrimination between the HCPs and LCPs.

**Figure 6:**
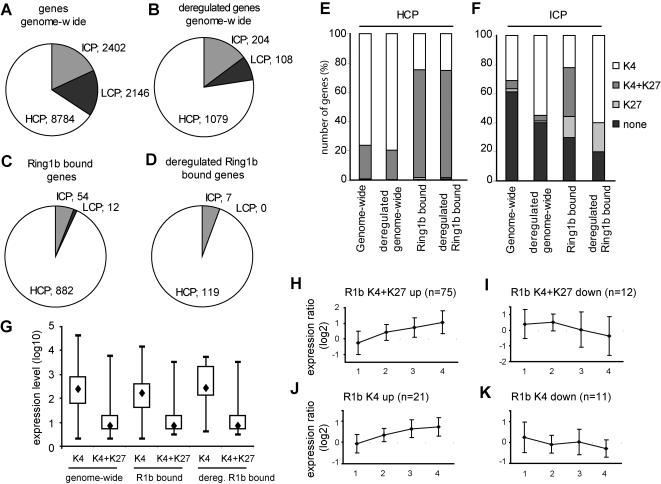
Deregulation of genes bivalent or H3K4me3 marked genes with HCPs in Ring1b-deficient ES cells. (A–D) Circle graphs show that Ring1b primarily occupies (C) and regulates (D) transcription of genes with high CpG content promoters (HCPs) rather than intermediate (ICP) or ‘low’ (LCP) CpG content promoters, compared to the distribution of HCPs in all genes (A) or only the deregulated genes (B) at the microarray (‘genome-wide’). (E) Bar graph showing the distribution (percentages) of the chromatin state of HCPs for all genes on the whole microarray (‘genome-wide’); only the genes deregulated in absence of Ring1b; Ring1b bound genes; only deregulated Ring1b bound genes in Ring1b-deficient ES cells. Graph shows that Ring1b binds and regulates transcription of bivalent, and to a lesser extend H3K4me3 marked genes (final two columns), in contrast to the genome-wide distribution of these marks on promoters (first two columns). (F) as (E), but for genes with ICPs. Graph shows that bivalent Ring1b bound genes with ICPs are not deregulated in Ring1b-deficient ES cells. (G) Blotplot representing Affimetrix array data of RNA expression levels in wild type ES cells [30], for the genes represented in (E). The blotplot shows that the median expression levels of H3K4me3 marked genes on the whole microarray (‘genome-wide’ ) are higher compared to the median RNA expression levels of H3K4me3+H3K27me3 marked genes (first two columns). This is similar for genes that are bound by Ring1b (middle two columns), and for Ring1b bound genes that are deregulated in Ring1b-deficient ES cells (last two columns). Black diamond represents median RNA expression level. (H–K) Line graph showing changes (log2 ratio) in gene expression of bivalently (H–I) or H3K4me3 (J–K) marked genes bound by Ring1b following deletion of Ring1b in *Ring1b^−/Lox^;CreER^T2^ ES* cells. Time is indicated in number of days of 4-OHT treatment.

Comparative analysis showed that about two-third of the promoters genome-wide, i.e represented on our microarrays, or differentially regulated in Ring1b-deficient ES cells, had HCPs (Figure 6A, 6B). Remarkably, Ring1b almost exclusively associated with and regulated transcription of genes with HCPs (93%; 882/948 and 94%; 119/126, respectively; Figure 6C, 6D). This suggests that Ring1b primarily regulates transcription of CpG-‘rich’ promoters.

We next analyzed the chromatin state of the Ring1b bound genes with HCPs. We found that these genes were strongly associated with bivalent marks (74%; 653/882). A similar fraction of deregulated Ring1b bound genes with HCPs was bivalently marked (73%; 87/119; Figure 6E). These deregulated bivalently marked Ring1b bound genes included mostly developmental genes, such as *Bin1*, *Bmi1*, *Bmp7*, *Col4a2*, *Ccnd2*, *En1*, *Gata3*, *Lmx1a* and *Wnt6*, and were predominantly derepressed in absence of Ring1b (86%; 75/87; Figure 6H, 6I). Interestingly, we found that Ring1b was also associated with a considerable number of HCPs with the H3K4me3 mark alone (25%; 218/882; Figure 6E). The lack of repressive H3K27me3 marks at these genes, catalyzed by PRC2, implies that Ring1b might be recruited independent from PRC2 for these genes in ES cells. Of the deregulated H3K4me3 marked Ring1b bound genes with HCPs 66% (21/32) was derepressed, including *Eif4g3*, *Gja1* and *Sgce*, and various histone encoding genes, such as *Hist1h2bn*, *Hist1h2bf*, *Hist1h3i*, *Hist1h4h*, and *Hist1h4f* (Figure 6J, 6K). Not all promoters associated with the active H3K4me3 mark are actively transcribed [30]. Considering the fact that Ring1b is a transcriptional repressor, we predicted that the H3K4me3 marked genes with relative low expression levels were bound by Ring1b. We therefore examined the RNA expression levels of these genes in wild type ES cells by using the Affimetrix RNA expression data that was available in the chromatin-state mapping study by Mikkelsen et al. [30]. Unexpectedly, Ring1b binding did not correlate with low expression levels of H3K4me3 marked genes, but instead seemed to associate with these actively marked genes indifferent of their level of expression (Figure 6G). Notably, in more committed NPCs and MEFs (89% and 83%, respectively) most of the Ring1b bound H3K4me3 marked genes retain this histone mark and corresponding transcriptional state, according to the chromatin-state maps generated for these cell types [30] (Figure S3). In contrast, of the bivalently marked genes only 11% and 51% retain both histone marks in NPCs and MEFs, respectively [30] (Figure S3), indicating that Ring1b does not act to silence the H3K4me3 marked genes in ES cells or during development. Although we cannot exclude that differentiation-associated changes affect transcription of the Ring1b bound genes, these data suggest that Ring1b predominantly represses bivalent genes, and is also involved in modulating, rather than silencing, the transcriptional activity of genes with the active H3K4me3 histone mark in ES cells.

Strikingly, a specific subset of Ring1b bound genes was not affected following Ring1b deletion. This subset contains LCPs, which were all without H3K27me3 or H3K4me3 marks, and bivalently marked genes with ICPs (Figure 6D, 6F). This implies that other mechanisms are involved in regulation of these genes in ES cells.

Summarizing, these data suggest that Ring1b is involved in direct regulation of RNAPII controlled promoters with ‘high’ CpG content and epigenetically distinct histone marks, namely bivalent marks or the active H3K4me3 mark, in mouse ES cells.

## Discussion

To investigate the role of Ring1b in mouse ES cells, we employed a conditional knockout system. By performing microarray analysis, we studied the genome-wide changes in gene transcription that occur following Ring1b deletion in ES cells. We found that in Ring1b-deficient ES cells key developmental genes were derepressed, including a subset of genes bound by stem cell regulators Oct4 and Nanog, suggesting that Ring1b deficiency results in activation of the differentiation program. Accordingly, specific differentiation-related pathways were differentially deregulated, including TGFbeta signaling, cellular communication and cell cycle pathways. Importantly, retained expression of stem cell regulators Oct4 and Nanog indicates that Ring1b-deficient ES cells still have important ES cell specific characteristics. Based on re-analysis of previously published global binding studies performed in mouse ES cells, we found that Ring1b predominantly binds and represses genes with CpG-‘rich’ promoters. Furthermore, these genes are associated with bivalent domains or the H3K4me3 histone mark alone. We suggest that Ring1b contributes to stable maintenance of pluripotency of ES cells by repressing bivalently or actively marked genes.

We found that Ring1b is required to repress the differentiation program in ES cells, which is consistent with the observation that Ring1b-deficient ES cells are prone for differentiation [34]. Comparative analysis with genome-wide Ring1b binding data of mouse ES cells [21], indicated that Ring1b is involved in direct transcriptional regulation of key developmental genes, such as *Bmp7*, *Gata3* and *Msx*. This is in agreement with other genome-wide and candidate-based PcG binding studies performed in mouse and human ES cells [21], [24]–[28], [34]. Notably, by directly repressing these extracellular and down-stream effector components of differentiation-related pathways, such as *Bmp7* and *Id3* of the Bmp/TGFbeta signaling pathway, and *Col4a2*, *Gja1* and *Ccnd2* of the cellular communication pathways, Ring1b may have an important role in influencing lineage choice and other developmental events. For example, expression of ECM protein Col4a2 was shown to induce mesodermal differentiation of ES cells *in vitro*, while repression of Bmp7 at the dorsal site of zebrafish gastrula was shown to allow cellular migration as is required for limb development. [52], [53]. In Ring1b knockout embryos, several developmental markers are misexpressed at early gastrulation stages, extraembryonal mesoderm is over-represented, epiblast has expanded improperly, and embryonic growth is delayed [17]. This is in line with the aberrant expression of developmental markers and induction of apoptosis observed in Ring1b knockout ES cells. The early embryonic lethality is partially rescued in a Cdkn2a-knockout background, one of the PcG transcriptional targets [54], [55], resulting in the delay of embryonic death of about 4 days [17]. Since relief of the Ink4a/Arf-mediated antiproliferative block only bypasses the proliferative defects induced by loss of Ring1b, this underscores the importance for Ring1b in regulating developmental genes during early embryogenesis.

The observation that only a subset of Ring1b bound genes is derepressed following deletion of Ring1b in ES cells, suggests that additional mechanisms mediate silencing of the other Ring1b target genes. This would be in correspondence with observations by others, who show that only part of the PcG bound genes are deregulated after RNAi-mediated depletion of PcGs in human embryonic fibroblasts and in ES cells deficient for Eed or Suz12 [20], [21], [26]. Partial derepression could be also be explained by functional redundancy by related Ring1a, which possess ubiquitin E3 ligase activity towards histone H2A, alike Ring1b, although with less efficiency [56]–[58]. In addition, the composition of PRCs can vary per tissue and developmental stage [20], [27], [56], [59]. Therefore, Ring1b might not be essential in every promoter-bound PcG repressive complex.

We found that Ring1b represses genes that are co-occupied by Oct4 and Nanog. This is consistent with earlier reports, which show a role for PcG proteins in repressing a set of developmental genes that are bound by Oct4 and Nanog in ES cells [25], [51]. A possible link between these proteins is provided by Wang and colleagues, who co-purified Oct4, Yy1 and Ring1b with stem cell specific protein Rex-1 from ES cells [60]. In *Drosophila,* PcG-specific sequences (named polycomb group responsive elements or PREs) mediate PcG repression [61]. However, in mammalians, PREs or sequence-specific DNA binding PcG proteins have not been identified. Therefore, one might speculate that recruitment of PcG-silencing complexes to specific genes involves interactions with site-specific DNA-binding factors. This could be the recruitment of the PcG silencing complex to key developmental genes through interaction with stem cell specific factors Rex-1 and/or Oct4 in order to maintain an undifferentiated state of ES cells. This suggestion is supported by the observation that Suz12 dissociates from PcG/Oct4 co-occupied promoters upon downregulation of Oct4 [20]. Interestingly, others have found that histone H2A E3 ligase 2A-HUB, but not RING1B, is selectively required for H2A mono-ubiquitination and subsequent repression of chemokine genes following recruitment by co-repressor N-CoR in human monocytes [32]. Together, this suggests that different site-specific factors are involved in the recruitment of distinct H2A E3 ligases to mediate repression of a specific set of genes.

Ring1b-deficient ES cells retain expression of important stem cell regulators Oct4, Nanog and alkaline phosphatase, which was also observed in other PcG-deficient ES cells [20], [31], [34]. Derepression of developmental genes and downregulation of other stem cell markers, such as *Rex-1*, *Sox2* and *Dppa3,* suggests activation of the differentiation program. During ES cell differentiation, repression of Oct4 and Nanog is essential, considering that ectopic expression of Oct4 can block differentiation, while expression of Nanog is enough to maintain ES cell self-renewal and pluripotency, even in the absence of extrinsic factor LIF or Oct4 expression [44], [45], [62]. Retained expression of Oct4 and Nanog could indicate that PcG proteins are either directly or indirectly involved in regulation of Oct4 and Nanog. A direct role for PcG proteins in regulating the expression of Oct4 and Nanog has been suggested by Pasini and co-workers, who show that PRC2 proteins bind to the Oct4 and Nanog promoters in ES cells [20]. Moreover, the H3K27me3 histone mark has been found at the Oct4 promoter in more committed cells, suggesting that PcG proteins are involved in direct repression of stem cell regulators in differentiated cells [28].

Comparative analysis of our expression profiling data with recently published global chromatin-state maps generated of mouse ES cells showed that Ring1b almost exclusively binds to genes with CpG-rich promoters. This is in consistent with a report showing that a strong correlation exists between promoters with highly conserved large CpG islands and Suz12 binding domains in undifferentiated human ES cells [63]. Furthermore, our data confirm that PcG proteins are involved in repressing bivalently marked developmental genes in ES cells [28]–[31]. This comparative analysis also revealed that a considerable number of actively transcribed H3K4me3 marked Ring1b bound genes are regulated (mainly repressed) in ES cells. The absence of H3K27me3 at these promoters suggests that Ring1b is recruited independently from PRC2. This is in agreement with other observations indicating that *Xist* RNA, but not PRC2, is required for PRC1 recruitment during X inactivation in differentiating ES cells [10]. The association of Ring1b to actively transcribed genes is consistent with global and candidate-based binding studies that show overlap between PcG and RNAPII occupancy or association of PcG proteins with expressed genes in mouse and human ES cells, neural progenitor cells and embryonic fibroblasts [20], [25]–[27], [64]. This suggests that Ring1b does not function as a silencer of these genes in ES cells. Moreover, a large subset of actively marked genes retain this mark and corresponding transcriptional state in more committed NPCs and MEFs [30]. Thus, rather than poising for activation or prolonged inactivation, as was proposed for the bivalent genes, Ring1b might be required to modulate the transcriptional activity of this subset of actively marked genes in ES cells and during later developmental stages. Only recently, association of Ring1b and uH2A was shown to mediate a silenced state of RNAPII present at promoters and coding regions of bivalent genes [31]. Dissociation of Ring1b and uH2A from these promoters results in activation of the RNAPII complex without major changes in the levels of PRC2 proteins or association of H3K27me3. In addition, others showed that the presence of uH2A in the promoter-proximal region blocks recruitment of FACT resulting in pausing of RNAPII and inhibition of transcriptional elongation [32]. One could speculate that Ring1b-mediated mono-ubiquitination of histone H2A also interferes with the RNAPII complex at active H3K4me3 marked genes, but how remains to be investigated.

Altogether, our data suggest that Ring1b is important to maintain an undifferentiated state of ES cells through repression of key developmental genes. The finding that this involves direct transcriptional repression of at least two epigenetically distinct sets of genes helps to understand and further explore the role of Ring1b during development.

## Materials and Methods

### Generating *Ring1b* conditional knockout ES cells

The *Ring1b* wild type allele in *Ring1b^+/−^* ES cells [17] was targeted with a construct containing loxP sequences at the EcoRI site in intron 2 and at the XhoI site in intron 4, followed by a third LoxP site enclosing a hygromycin cassette to allow for selection of targeted ES cells. Correct targeting of hygromycin resistant *Ring1b* ^−*/LoxHyg*^ ES cell clones was confirmed by Southern blot analysis. Next, to obtain *Ring1b^−/Lox^* ES clones the hygromycin cassette was removed by transiently expressing adenoviral Cre and subsequently screening for hygromycin sensitivity. Correct removal of the hygromycin cassette in the selected *Ring1b^−/Lox^* ES clones was confirmed by Southern blot analysis. Finally, to generate inducible conditional *Ring1b^−/Lox^;CreER^T2^* ES cells a puromycin-selectable R26CreER^T2^ construct was targeted to the ROSA26 locus. CreER^T2^ could be activated by adding 200 nM 4-OHT (Sigma, dissolved in absolute ethanol) to the ES cell medium.

Southern blot analysis of HindIII digested genomic DNA and a 5′end external probe in intron 1 to discriminate between the *Ring1b* wild type (11 kb), conventional knockout (5 kb)[17], *Lox* (7 kb), and *Del* (4 kb) allele. The *Del* allele follows from deletion of exons 3 and 4 after 4-OHT-mediated activation of CreER^T2^ in *Ring1b^−/Lox^;CreER^T2^* ES cells.

### Cell culture

ES cells were cultured on dishes coated with 0.1% gelatin (Sigma) without feeders in 60% conditional Buffalo Rat Liver (BRL) medium, which consisted of 60% BRL medium and 40% standard ES medium. Standard ES cell medium consisted of GMEM (Gibco), 10% foetal calf serum (Sigma), supplemented with 1% non-essential amino acids, 1 mM sodium pyruvate, L-glutamate, 0.1 M beta-mercaptoethanol and 10^3^ units LIF (all Gibco). BRL medium was derived by filtering (0.2 uM) the supernatant of BRL cells cultured for one week in standard ES cell medium without beta-mercaptoethanol or LIF.

*Ring1b* conditional ES cells or *Ring1b^+/+^;CreER^T2^* control ES cells were treated with 200 nM 4-OHT and MEFs with 1 µM 4-OHT for the indicated time. MEFs were isolated from 14.5 day old embryo's and cultured as described before [55]. For RA treatment ES cells were cultured in standard ES medium without LIF and in the presence of 500 nM all-trans-RA (ATRA) (Sigma).

### Protein isolation and Western blot analysis

Proteins were extracted from whole cells (RIPA lysates, protocol as described previously) [65] or from the nucleus (nuclear extraction). For nuclear extracts cells were washed with PBS, incubated on ice for 5 min in Triton lysis buffer (0.1 M NaCl, 0.3 M sucrose, 3 mM MgCl2, 50 mM HEPES pH 6.8, 1 mM EGTA, 0.2% Triton-X100) supplemented with Complete protease inhibitor cocktail and 10 mM N-ethylmaleimide (NEM) followed by spinning at for 5 min 3000 rpm at 4°C to pellet the nuclei. Nucleic proteins were recovered by incubating the nuclei in SDS-lysis buffer (0.1% SDS, 50 mM TrisHCl pH 7.5, 0.15 M NaCl) for 10 min on ice, followed by sonification (10 pulses at 50%, level 4) and spinning for 1 min at 14.000 rpm to remove insoluble material. Protein samples were assayed with SDS-PAGE using pre-cast gradient gels (Invitrogen) and conventional Western blotting techniques.

### Immunostaining, alkaline phosphatase, and Annexin V staining

ES cells were cultured four days with 200 nM 4-OHT or three days with 500 nM RA on coverslips coated with 0.1% gelatin. Cells were fixed in 4% paraformaldehyde for 10 min at RT and permeabilization in 0.25% Triton-X100/PBS for 5 min. Cells were then incubated for 30 minutes at RT in blocking solution (5% foetal calf serum (Gibco), 5% normal goat serum (Vector laboratories), 0.02% Triton-X100/PBS). Next, cells were incubated with the 1^st^ antibody for 1 hour at RT in blocking solution and afterwards washed five times 5 minutes with 0.02% Triton-X100/PBS. Hereafter, cells were incubated for 1 hour at RT with fluorescence labeled 2^nd^ antibody in blocking solution, followed by five washes of 5 min each with 0.02% Triton-X100/PBS. Cover slips were mounted on object slides using Vectashield with DAPI (Vector Laboratories). For the uH2A immunostaining cells were permeabilized prior to fixation by in cytoskeletal buffer (100 mM NaCl, 300 mM sucrose, 3 mM MgCl2, 10 mM PIPES pH6.8) for 30 sec, followed by 30 sec in cytoskeletal buffer containing 0.5% Triton-X100, and 30 sec in cytoskeletal buffer without Triton-X100, all on ice.

For the alkaline phosphatase staining cells were cultured on dishes, followed by fixation and stained according to the manufactures protocol (Sigma).

To determine the level of apoptosis, cells were collected by trypsinization, or first washed twice with PBS to obtain only the adherent cells, followed by staining with FITC-conjugated Annexin V-FITC (Roche) according to the manufactures protocol and analyzed by flow cytometry.

### Antibodies

H3K27me3 rabbit polyclonal was a kind gift from T. Jenuwein. Ezh2 mouse monoclonal antibody was a kind gift from K. Helin. Ring1b mouse monoclonal and rabbit polyclonal antibodies were obtained from H. Koseki. Oct4 mouse monoclonal antibody C-10 and Lamin-B goat polyclonal antibody M20 from Santa Cruz, Nestin mouse monoclonal antibody from BD, Eed rabbit polyclonal and Bmi1 and uH2A mouse monoclonal antibodies from Upstate. Secondary immunofluorescence antibodies were Alexa 488- or 568-conjucated anti-mouse, 568- or 488-conjucated anti-rabbit from Molecular Probes.

### Image analysis and quantification

To quantify the fluorescence levels of immunofluorescence-labeled proteins in individual cells, we took sequential images of DAPI and the fluorescence labeled protein using a Leica confocal research microscope with a 63x lens and fixed laser settings per protein. Files were saved in TIFF format with 512×512 resolution. The images were analyzed with Image-Pro 5.1 software (Media Cybernetics). In brief, mean fluorescence intensity levels of the immunofluorescence-labeled protein per cell were measured for the nucleic area as was determined by the DAPI staining for on average 250 cells per quantification and three independent stainings. The distribution of the mean intensity per cell is displayed in histogram figures with same x-axis scale.

### RNA isolation, real-time QPCR and microarray analysis

*Ring1b^−/Lox^;CreER^T2^* and *Ring1b^+/+^;CreER^T2^* control ES cells (used as a reference for QPCR and microarray) were cultured under the same conditions and RNA was isolated at the indicated time points. Total RNA was isolated using TRIzol reagent (Invitrogen) according to the manufactures protocol followed by DNase treatment and aRNA amplification (Invitrogen). Real-time QPCR conditions and primer sequences are as described elsewhere [21]. Changes in RNA levels following 4-OHT treatment of *Ring1b^−/Lox^;CreER^T2^* relative to *Ring1b^+/+^;CreER^T2^* ES cells were determined based on the results of three QPCRs.

The oligonucleotide arays were printed at the CMF core facility of the NKI/AvL with mouse Operon microarray version 3 (http://microarrays.nki.nl/download/geneid.html). Detailed information about the labeling and hybridization protocols and array analysis can be found on the NKI/AvL microarray website (http://microarrays.nki.nl/download/protocols.html). In brief, amplified RNA was labeled with ULS Cy5 or Cy3 (Kreatech EA-006, Amsterdam) was pooled and co-hybridized to the microarray (each experiment was performed in dye swap fashion and controlled with self-self hybridization experiments to correct for dye-bias). After 16 hours of hybridization the microarrays were washed and scanned using an Agilent DNA Microarray scanner (Agilent Technologies, Cat# G2505B). ImaGene v6.0 software (BioDiscovery Inc) was used to quantify the RNA expression levels using the tiff images produced by the scanner. The background-corrected intensities from the Cy5- and Cy3 channel were used to calculate log2 transformed ratios. These ratios were normalized using a lowest fit per subarray [66]. A weighted average ratio and confidence level (P-value) was calculated per gene by a NKI platform adjusted error model [67], which was fine-tuned by self-self hybridizations. Significantly differentially expressed genes (outliers) between sample and reference were selected based on their P-value (a gene with a P-value <0.05 was considered an outlier).

The microarray details and results are submitted to the ArrayExpress database (http://www.ebi.ac.uk/arrayexpress/), reference number E-NCMF-14. Clustering of the microarray data was performed using the bioinformatics software program Genesis [68]. GO classification analysis was performed using bioinformatics program BiNGO [36]. Pathway analysis was performed using the bioinformatics software program DAVID based on the KEGG pathway database available online (http://david.abcc.ncifcrf.gov).

## Supporting Information

Figure S1 Loss of uH2A after deletion of Ring1b in MEFs. Example of immunofluorescence stainings of Ring1b-/Lox;CreERT2 MEFs 6 days following 4-OHT treatment showing loss of uH2A in MEFs that have lost Ring1b.
(4.21 MB TIF)

Figure S2 Graphical representation of GO categories that are significantly enriched in Ring1b-deficient ES cells. Graphical map showing the hierarchical relations between the significantly enriched GO categories (yellow circles) identified by the BiNGO bioinformatics tool based on the outliers per day of 4-OHT treatment of Ring1b-/Lox;CreERT2 ES cells.
(0.32 MB PDF)

Figure S3 Ring1b bound H3K4me3 marked genes with CpG-rich promoters retain this mark in NPC and MEFs. Bar graph showing the distribution of the chromatin-state of the Ring1b bound genes with high-CpG content promoters (HCP) in neural progenitor cells (NPC) or MEF that were marked with either H3K4me3 or H3K4me3+H3K27me3 in ES cells. Graph represents the chromatin state of all Ring1b bound genes that are represented on the microarray (‘all’), and only the Ring1b bound genes that are deregulated in Ring1b deficient ES cells (‘dereg’). These data show that most H3K4me3 marked genes, retain this mark in NPC and MEFs.
(0.59 MB TIF)

Table S1 Expression Profiling data of Ring1b-deficient ES cells. Excel file of expression profiling data of Ring1b-deficient ES cells, coupled to Ring1b binding data according to Boyer and co-workers (Nature, 2006), based on EntrezIDs.
(7.49 MB XLS)

Table S2 BiNGO ontology analysis of genes deregulated in ES cells. Excel file of BiNGO analysis of all outliers based on EntrezIDs per day of 4-OHT treatment of Ring1b-/Lox;CreERT2 ES cells.
(0.06 MB XLS)

Table S3 Pathway analysis of genes transcriptionally deregulated following Ring1b deletion. Excel file of DAVID pathway analysis (selecting the KEGG pathway database option) of all outliers, based on EntrezIDs, per day of 4-OHT treatment of Ring1b-/Lox;CreERT2 ES cells.
(0.02 MB XLS)
