# Supplementary figures and images for: Ubiquitin E3 Ligase Ring1b/Rnf2 of Polycomb Repressive Complex 1 Contributes to Stable Maintenance of Mouse Embryonic Stem Cells
Source: PLoS One. 2008 May 21;3(5):e2235. doi: 10.1371/journal.pone.0002235 (PMC2375055; doi:10.1371/journal.pone.0002235)

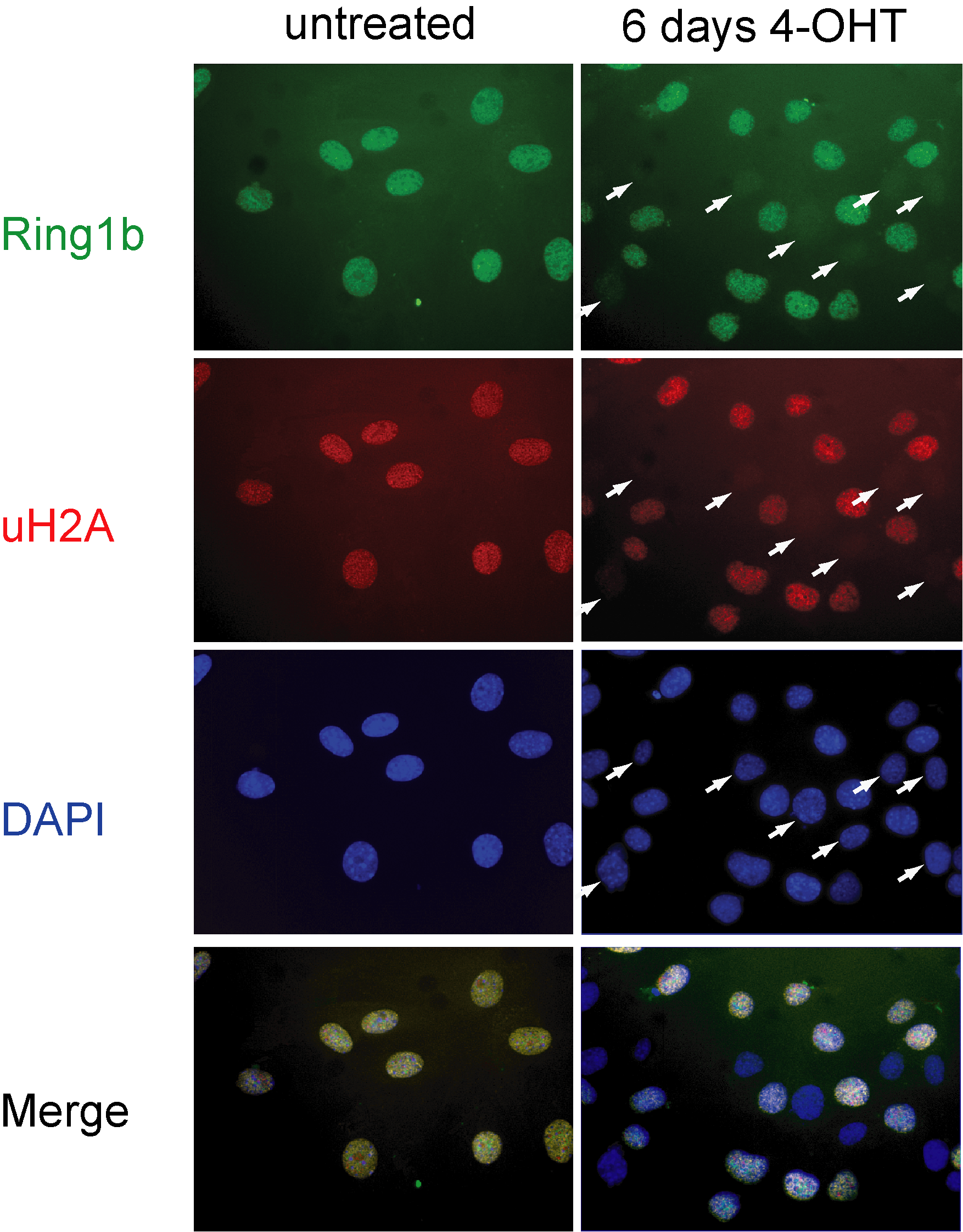

Supplement: Figure S1 — Loss of uH2A after deletion of Ring1b in MEFs. Example of immunofluorescence stainings of Ring1b-/Lox;CreERT2 MEFs 6 days following 4-OHT treatment showing loss of uH2A in MEFs that have lost Ring1b. (4.21 MB TIF) [file pone.0002235.s001.tif]

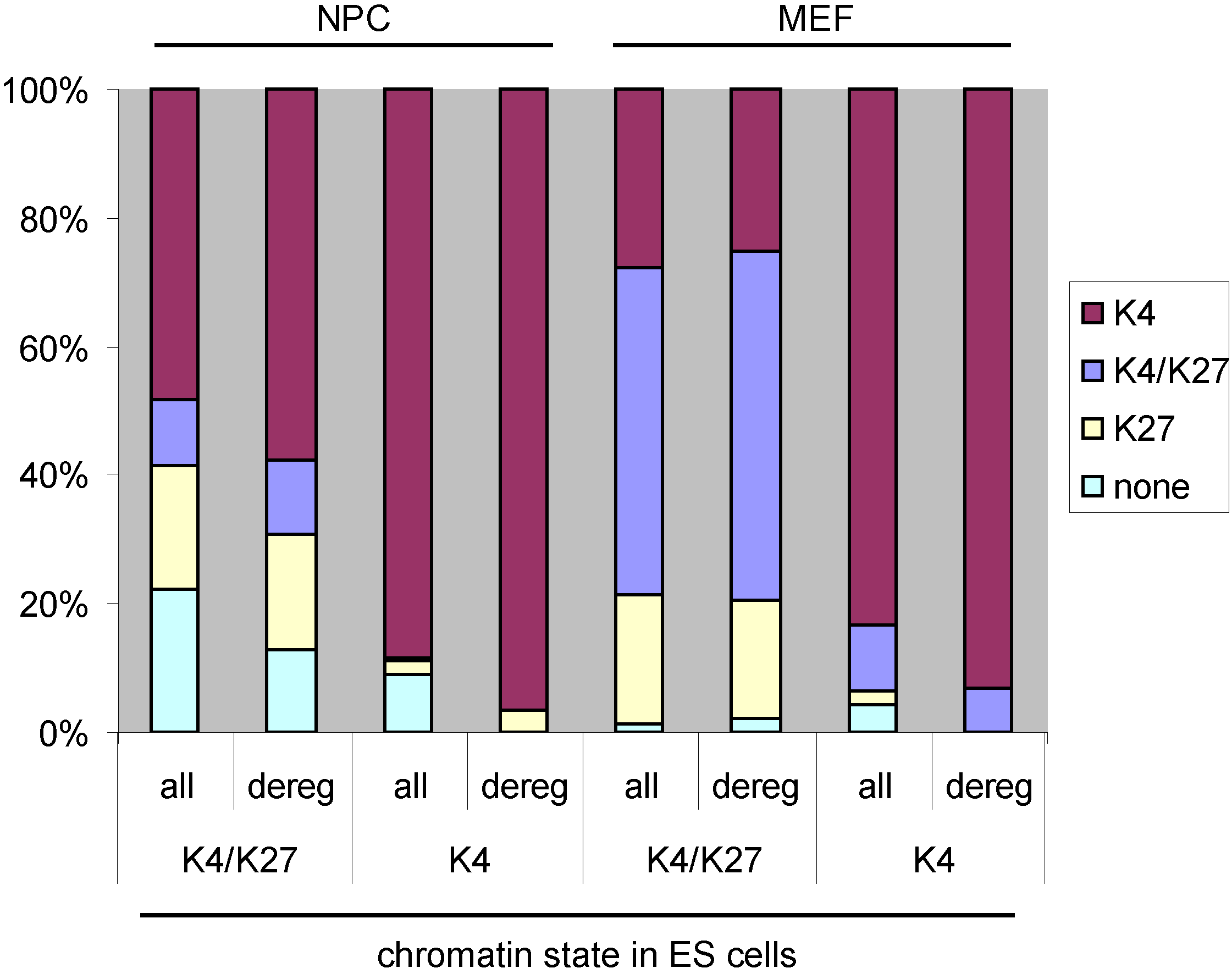

Supplement: Figure S3 — Ring1b bound H3K4me3 marked genes with CpG-rich promoters retain this mark in NPC and MEFs. Bar graph showing the distribution of the chromatin-state of the Ring1b bound genes with high-CpG content promoters (HCP) in neural progenitor cells (NPC) or MEF that were marked with either H3K4me3 or H3K4me3+H3K27me3 in ES cells. Graph represents the chromatin state of all Ring1b bound genes that are represented on the microarray (‘all’), and only the Ring1b bound genes that are deregulated in Ring1b deficient ES cells (‘dereg’). These data show that most H3K4me3 marked genes, retain this mark in NPC and MEFs. (0.59 MB TIF) [file pone.0002235.s003.tif]
