# Supplementary material for: Ubiquitin E3 Ligase Ring1b/Rnf2 of Polycomb Repressive Complex 1 Contributes to Stable Maintenance of Mouse Embryonic Stem Cells
Source: PLoS One. 2008 May 21;3(5):e2235. doi: 10.1371/journal.pone.0002235 (PMC2375055; doi:10.1371/journal.pone.0002235)

BiNGO analysis with outliers at day 1

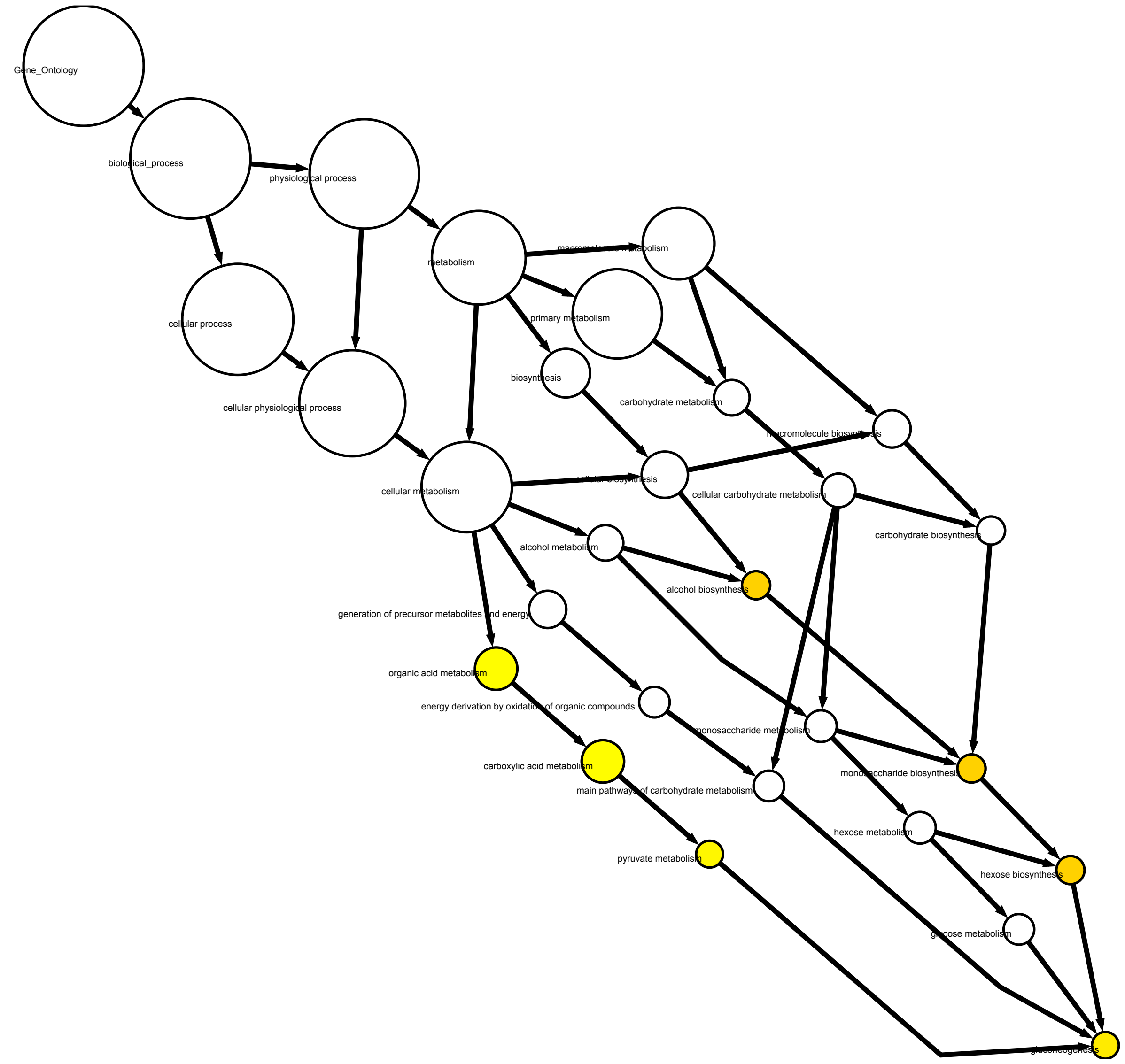

BiNGO analysis with outliers at day 2

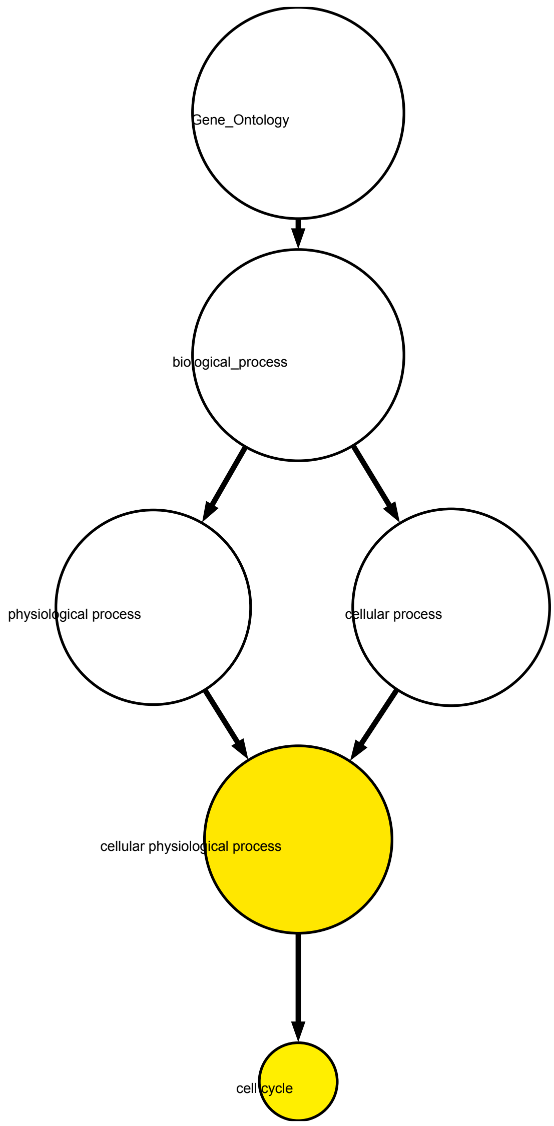

BiNGO analysis with outliers at day 3

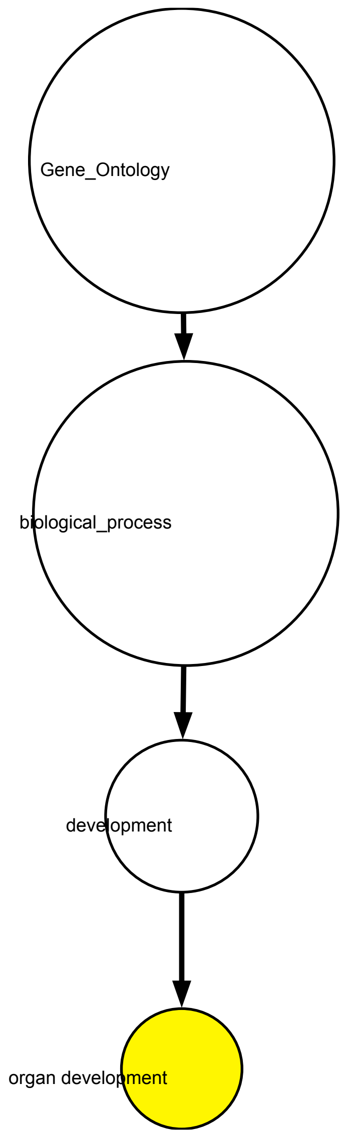

BiNGO analysis with outliers at day 4

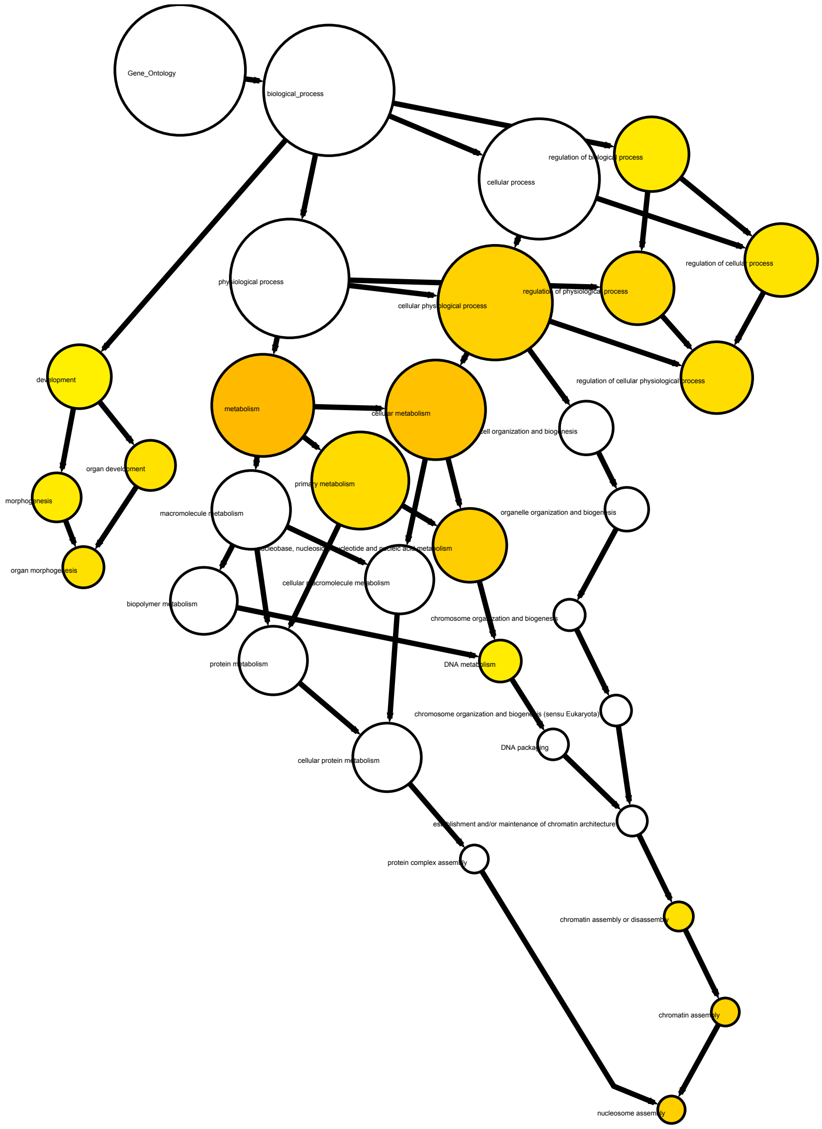

Supplement: Figure S2 — Graphical representation of GO categories that are significantly enriched in Ring1b-deficient ES cells. Graphical map showing the hierarchical relations between the significantly enriched GO categories (yellow circles) identified by the BiNGO bioinformatics tool based on the outliers per day of 4-OHT treatment of Ring1b-/Lox;CreERT2 ES cells. (0.32 MB PDF) [file pone.0002235.s002.pdf]
